# How does parents’ social support impact children’s health practice? Examining a mediating role of health knowledge

**DOI:** 10.1186/s41256-023-00291-5

**Published:** 2023-03-21

**Authors:** Paulin Tay Straughan, Chengwei Xu

**Affiliations:** 1 School of Social Sciences, Singapore Management University, Singapore, Singapore; 2 Public Management and Policy Analysis (PMPP), Graduate School of International Relations (GSIR), International University of Japan (IUJ), Minamiuonuma, Japan

**Keywords:** Childhood obesity, Health knowledge, Health practice, Social support, Social determinants, Weight management

## Abstract

**Background:**

Family environmental factors play a vital role in shaping children’s health practices (e.g., obesity prevention). It is still unclear how parents’ social support affects children’s obesity-related health practices. The present study argues that whether parents’ social support positively associates with children’s obesity-related health practice depends on if it could promote parents’ obesity-related health knowledge. Thus, we hypothesize that health knowledge mediates the relationship between parents’ social support and children’s health practice regarding weight management.

**Methods:**

To test the hypothesis, we conducted a questionnaire survey and collected a nationally representative sample of 1488 household responses in Singapore. The survey included questions about parents’ social support, health knowledge, children’s health practices, and socio-demographic variables. All participants have at least one child 14 years old or younger. In the sample, 66.1% of the respondents are female, and 93.7% are below 50 years old. Structural equation modeling (SEM) via Stata was used to examine the associations between parents’ social support, health knowledge, and children’s health practice.

**Results:**

The results of our analysis support our hypothesis. Specifically, (1) parents’ social support shows a positive relationship with health knowledge (Coef. = 0.17, *p* < 0.001 for BMI knowledge and Coef. = 0.18, *p* < 0.001 for nutrition knowledge); (2) parents’ social support (total effect of social support = 0.081, *p* = 0.071) and health knowledge positively associate with children’s obesity-related health practice (coefficient of BMI knowledge = 0.10, *p* < 0.01; coefficient of nutrition knowledge = 0.31, *p* < 0.001); and (3) the effects of parents’ social support on children’s health practice is fully mediated by parents’ health knowledge (mediating effect = 100%, *p* = 0.007).

**Conclusion:**

The present study provides fresh evidence from a multicultural context to understand the relationships between parents’ social support, health knowledge, and children’s obesity-related health practice. Our findings support the argument that social support from parents’ social networks does not necessarily promote health outcomes. The only social support that carries proper health knowledge can facilitate good health practice.

## Background

The increasing prevalence of childhood obesity has been one of the most challenging issues faced by both developed and developing countries [1]. Between 1980 and 2013, the rate of childhood overweight and obesity jumped from 16.6 to 23.2% in developed countries, and from 8.3 to 13.2% in developing countries [2]. In response to the heightened concern, parental influence over child weight merits attention. Many studies support the view that parents are highly responsible for childhood obesity and obesity prevention practices [3, 4]. Golan and colleagues even suggested that health promotion programs focusing on parents only are more effective than that involving both parents and their children with obesity [5]. How parental factors impacts children’s health practice merits attention. Notably, physical activity and food intake control have been identified as two critical means of intervention for parents to manage their children’s body weight. Thus, the present study focuses on children’s participation in physical activities and diet control as the primary obesity-related health practice.

Parents’ social support is critical for parenting practice, and whether social support has a positive impact depends very much on their social environment, in particular who parents can draw knowledge and advisories from [6, 7]. Social support for parenting comprises both formal and informal support. Formal support is conceptualized as caregiving help provided by professionals and formal organizations, where assistance is governed by contractual rather than affiliative norms [8, 9]. The operationalization of formal support typically determines if the care recipient and/or caregiver uses specific services. Typical services include home health, daycare, support groups, transportation, and referral services. In contrast, informal social support tends to be provided within an individual’s network, comprising mainly family and friends [10]. Yet, while the relationship between an individual’s social support and their health and weight management has been explored [6], it has not been explored as thoroughly with regards to parent’s child-rearing practices. That is, there is still a dearth of literature focusing on the effects of parents’ social support on their children’s obesity-related health practice (e.g., physical exercise and eating less junk food).

Further, whether social support always produces positive impacts is questionable. Specifically, there is a lack of empirical evidence to demonstrate what is likely to enable social support to positively impact health behaviors, and conversely what causes social support to fail to make a difference or even have a negative effect. Although many studies indicate a positive relationship between social support and health practice [11, 12], some scholars presented inconsistent findings [13]. Several studies indicate that social support can, in some cases, bring positive effects, and in other cases, negative effects on health behaviors, depending on whether social support carries salutary health-related knowledge or inadequate knowledge may have adverse health effects [14, 15]. We thus can speculate that health knowledge may mediate the relationship between social support and health practice. Put differently, the present study argues that the only social support that carries proper health knowledge can facilitate good health practice.

The primary hypothesis of this paper posits that the relationship between parents’ social support and children’s obesity-related health practice is determined by parents’ health knowledge of weight management (e.g., knowledge about body mass index and what constitutes a healthy diet). In particular, the mediating effect of health knowledge on the relationship between social support and health practice is to be examined. Health knowledge refers to parents’ general understanding and awareness of what constitutes obesity, how obesity is measured, what constitutes a healthy diet, and parents’ ability to comprehend nutritional labels on food products. We expect that parents’ social support will enhance children's health practice only when parents are knowledgeable about how to manage body weight. Hence, parents’ obesity-related knowledge may mediate the relationship between social support and health practice.

To examine the hypotheses, empirical evidence is drawn from a nationally representative survey of 1,488 parents with children who are 14 years old and younger in Singapore. We use structural equation modeling (SEM) to verify the hypotheses of the associations between parents’ social support, health knowledge, and children’s health practice. The following section reviews the existing literature and presents the study’s hypotheses. Next, we introduce data, analysis, and results. Lastly, we discuss the findings, implications, future directions, and conclusions.

## Literature review

### Social support and health practices

The effect of social support on health outcomes has been an important research topic for the past four decades [14]. Social support refers to support and resources that an individual can receive from his or her social networks (e.g., family members, friends, relatives, colleagues, and neighborhood residents) [16]. It can be given in the form of problem-solving information or advice, positive interactions, emotional or affective support, or even tangible aid [17, 18]. The concept is important for health outcomes as scholarship has shown that when people face health problems, they are very likely to seek support from people within their close networks [19].

Current research has indicated that there is a causal relationship between social support and positive health practices and outcomes [11, 12]. It has been argued that information and assistance from friends and relatives can promote patients’ awareness of seeking medical care [20]. For instance, one study found that women with obesity who received frequent support from family and friends had a higher possibility of losing weight [6]. Evidence from participants from 16 countries also indicated that low social support is associated with less physical activities [21].

**Fig. 1:**
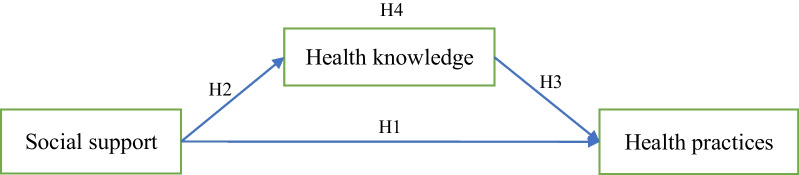
Hypotheses

Scholars have also linked parental social support to parenting practices and their children’s health outcomes. For example, social support from relatives and friends has significantly influenced parents’ parenting capacities and practices [17, 22]. Other studies have illustrated that parents with greater social support from their extended kin are more likely to have healthier children [23]. One of the possible mechanisms identified that enables this could be that support from family and friends helps parents start and maintain a healthier lifestyle with their children (e.g., regularly participating in physical exercises or eating less junk food) [24–26]. In light of this, we hypothesize that (see Fig. 1):

#### H1

Parents’ social support is positively associated with their children’s health practices.

### Social support and health knowledge

An important dynamic in the way social support influences health practice is the transmission of health knowledge. Despite the lack of consensus on the definition, health-related knowledge is a concept that is commonly agreed upon to be key within the health literacy framework [27]. Baker argues that health-related knowledge could facilitate the development of health literacy as it is with prior knowledge that the individual can comprehend health information and is, in that sense, literate [28]. This runs counter to frameworks developed by Nutbeam and colleagues, who deem health knowledge as an aspect of health literacy [27, 29]. They posit that health knowledge is necessary as it is what the individual acts upon to be considered health literate.

Social support serves as a critical means for individuals to gain health knowledge. Coleman argued that social support is important in gaining new information and serves as information channels, whereby the use of social relations with others provides the means through which one can acquire more knowledge. He further argued that while individuals may maintain social relations for other purposes, knowledge is also passed through in the process [30]. In House’s conceptualization of social support [31], informational support, or the provision of useful information, is one of the four forms of providing social support. Studies have also shown that individuals tend to seek health information from interpersonal sources as they may provide information tailored to their needs [32, 33]. A person’s social support may significantly impact his or her health knowledge. Therefore, we hypothesize that:

#### H2

Parents’ social support is positively associated with parents’ obesity-related health knowledge.

### Health knowledge and health practices

The impact of health knowledge on an individual’s health practice has been well explored. It has been argued that knowledge plays a major role in behavioral change [34]. Particular to health behavior, individuals with adequate health knowledge tend to adopt more preventive care [35–37]. A study based in Italy found that people with adequate nutrition knowledge are more likely to have healthier dietary patterns and a lower prevalence of obesity [38]. On the flipside, a lack of health knowledge has also been shown to lead to more health risk behaviors and poor health status [39, 40]. Studies found that the lack of health knowledge or literacy is associated with chronic diseases, higher rates of hospital admissions, longer hospital stays, and even unnecessary use of health care resources [41, 42].

However, the health knowledge of individuals is important not just for individuals’ own health outcomes, but in the case of parents, the health outcomes and practices of their children [43]. This is because parents play a dominant role in children’s lifestyles, particularly in the case of younger children in their formative years. It has been shown, for instance, that parents’ possession of health-related knowledge has positive effects on children’s health practice [44, 45]. Evidence also shows that children are more likely to have a better health status when their parents understand disease prevention [46] [. Concurrently, children whose caregivers had limited health literacy and less health knowledge tend to have worse health outcomes [47]. Yin and colleagues found that caregivers with inadequate health knowledge knew little about weight-based medication dosing and used non-standardized dosing instruments when administering medications [48]. Another study on children aged six and below showed that children whose caregivers with limited oral health knowledge tended to practice more harmful oral health behaviors, such as no daily cleaning or no brushing [49, 50]. Etelson and colleagues found that parents of children with excess weight are generally unable to recognize that their children have a weight problem [4]. And they argue that the success of any obesity prevention practices targeting young children depends on parents’ capability to recognize the overweight/obesity problem and to provide a healthy diet. Based on what we discussed above, we could argue that parents’ knowledge and capability to recognize obesity (e.g., identifying overweight/obesity) and to provide health interventions (e.g., providing a healthy diet) may significantly impact children’s health behaviors (e.g., participation in physical exercises). We thus hypothesize that:

#### H3

Parents’ obesity-related health knowledge is positively associated with their children’s health practice.

Despite this, several studies have also argued that social support can bring about negative health outcomes. Specifically, adequate information from individuals’ social networks may facilitate healthful knowledge and practice, whereas inadequate support or negative information may have an adverse health influence, especially for those with low health literacy [39, 51]. Thus, while there is strong evidence that positive social support has protective effects against all-cause mortality [14, 52], and that adequate resources help individuals to cope with health issues [39, 53], misleading information or advice, on the other hand, may hinder patients from seeking appropriate medical care or even reinforce unhealthy practice [15]. For example, for individuals with risky health practices (e.g., smoking and heavy drinking), social support from people with similar habits may normalize and maintain those unhealthy practices [39, 54]. Therefore, we can speculate that social support promotes health practice only when it can provide adequate health knowledge. It is not how much social support parents have in their child-raising endeavors but rather, what this support contributes to their health knowledge that matters. The relationships between social support, health knowledge, and health practice are further illustrated in Fig. 1.

Building on all the discussions above, we hypothesize that:

#### H4

The effect of parents’ social support on children’s health practices is mediated by parents’ obesity-related health knowledge.

## Methods

### Participants

Data is drawn from a nationally representative survey of parents with young children (age 14 years and younger) conducted in Singapore between June and November 2018. The sampling was based on a representative sample of household addresses provided by the Singapore Department of Statistics (SDOS). As requested by the research team, SDOS randomly drew 2116 household addresses from the total population excluding those without children or children aged 15 and above. Once we received the list of addresses, our research team proceeded to visit the 2116 households and conduct face-to-face surveys. The questionnaire includes measurement scales of social support, health knowledge, health practices, and socio-democratic variables. Each survey took about ten to 15 min to be completed. We prepared questionnaires in Chinese, English, Malay, and Tamil though all participants responded in English. Of the 2116 households being visited, 1488 valid responses were returned and the response rate was 70.6%. We conceptualized that parents’ influence is strongest when children are 14 years old or younger as parents remain socially significant in these children’s everyday lives. At this age, children tend to be homebound and are less likely to be influenced as strongly by peers and social media (compared to older teenagers, for example). Thus, the unit of analysis for our study was parents with a child age 14 years or younger. In the sample, 66.1% of the respondents are female, 30.1% are younger than 36 years old, 30.7% are between 36 and 40 years old, 33.0% are between 41 and 49 years old, and 6.3% are 50 years old and above; of the respondents, 40.4% have a bachelor’s degree, 33.3% have a post-secondary diploma, and 26.3% have secondary education and below. Among the respondents, 61.2% are Chinese, 20.2% are Malay, and 18.6% are Indian. This ethnic ratio is generally consistent with the country’s ethnic composition. More information about the sample profile is available in Table 1 which presents the frequencies of gender, age group, work status, housing type (as a proxy for social class), and education.

**Table 1:** Frequency table of gender, age group, work status, education, and residence type

Gender	Percent	Age	Percent
Female	66.11	30 and below	8.6
Male	33.89	31 to 35	21.47
Total	100	36 to 40	30.7
		41 to 49	32.97
		50 and above	6.26
		Total	100
**Work status**	Percent	**Education level**	Percent
Not working now	22.07	Below secondary	8.81
Working part-time	7.81	Secondary	17.48
Working full-time	70.12	Post-secondary (A levels and poly diploma)	33.33
Total	100	Bachelor and postgraduate	40.38
		Total	100
**Residence type**	Percent		
HDB 1-room to 3-room	21.28		
HDB 4- to 5-room	72.03		
Private apartment, condo or landed property	6.68		
Total	100		

*N* = 1488. *HDB* refers to the homes built by the Housing & Development Board (HDB) of Singapore

### Measures

*Social support.* To measure social support, Sarason and colleagues used a six-item index that operationalizes social support by counting the number of support sources [10]. Participants are asked to list the people whom they counted on to help them. A higher score indicated greater perceived availability of social support. Procidano and Heller employed a list of dichotomous items to count the number of support sources (e.g., My friends are good at helping me solve problems; 1 = yes, and 0 = No) [55]. Zimet and colleagues proposed a multi-dimensional scale that includes support from family, friends, and significant others (e.g., “My family really tries to help me”, “I can talk about my problems with my friends”, “There is a special person who is around when I am in need”, etc.) [56]. Building on the measurements developed in the above-mentioned studies, we employed five dichotomous items to measure the number of available support sources (see Table 2). Besides the two items on support from family and friends (e.g., “Do you have family members / close friends whom you trust to discuss childcare matters with?”), we also adapted Zimet and colleagues’ scale items of the support for significant other and created three new items: “Are you able to seek help from a doctor, when you need to?” and “Are you able to seek help from other health care providers like a nurse or dietitian, when you need to?”. Further, we include another item to capture parents’ general ability to look for help: “Do you know where to look for information on child nutrition and well-being?”. All indicators have a dichotomous outcome (1 = Yes and 0 = No). The number of ‘Yes’ answers is accumulated to create an index of social support.

**Table 2:** Measurement scale of social support, health knowledge, and health practices

Variables	Min	Max	Mean/percentage of positive response
*Social support *[55, 56, 59]			
Do you know where to look for information on child nutrition and well-being?	0	1	80.4%
Do you have family members whom you trust to discuss childcare matters?	0	1	88.8%
Do you have close friends whom you trust to discuss childcare matters?	0	1	78.9%
Are you able to seek advice from a doctor, when you need to?	0	1	90%
Are you able to seek advice from other health care providers like a nurse or dietitian, when you need to?	0	1	79.4%
*Health knowledge *[57, 58]			
*BMI knowledge*			
Do you know how obesity is measured?	0	1	66.8%
Do you know what is BMI?	0	1	87.3%
Will you be able to tell if your child is obese by checking your child’s height and weight?	0	1	58.7%
*Nutrition knowledge (of weight management)*			
I have a good knowledge of what constitutes a healthy nutritious diet for children	1	4	3.02
I know what my children should consume	1	4	2.96
Children should eat home-cooked meals instead of food bought from outside	1	4	3.31
I do not understand the details printed on nutrition labels (reversed)	1	4	2.89
I read the nutrition labels on the food products	1	4	2.93
I know how to help my child stay within the acceptable weight range	1	4	3.05
*Health practice*			
How often do your children exercise?	1	5	2.64
How often do your children eat out at a fast food restaurant? (reversed)	1	5	3.05

*Obesity-related health knowledge. *The present study assesses two aspects of health knowledge: knowledge on what constitutes obesity (e.g., knowledge of BMI) and knowledge on nutrition (e.g., what constitutes a healthy diet). Although many scales have been developed to measure disease-related knowledge, few studies address the measurement of knowledge about bodyweight management (e.g., knowledge of BMI and healthy diet). One study assessed nutrition knowledge by four items (e.g., Knowledge of recommended fruit servings a person should eat each day) [57]. Carter et al. measured patients’ cancer knowledge using a seven-item scale (e.g., “Do you know what breast cancer is?” “Do you know what a mammogram is?” [58]. Building on Carter and colleagues’ work, we assess BMI knowledge with three items (e.g., “Do you know how obesity is measured?”, “Do you know what is BMI?”, and “Will you be able to tell if your child is obese by checking your child’s height and weight?”). For knowledge of nutrition, we constructed six Likert scale instruments that captured respondents’ understanding of what constitutes a healthy diet and their confidence that they could provide for the nutritional needs of their children (see Table 2).

*Obesity-related health practice*. We use two items to capture weight management practices: “How often do your children exercise?” and “How often do your children eat out at a fast food restaurant?” Both items were rated by five-point-Likert scale items (from 1 = ‘Rarely or never’ to 5 = ‘Daily’). These items capture the frequency of health practices, which is the main weight control practice for children, and manifest both parents’ and children’s proactive role in weight management.

*Control variables. *To improve the robustness of the structural equation model, we include several control variables in the analysis. Parents’ social-economic status has been shown to play a significant influence on parenting practice and children’s participation in physical activities and eating [60, 61]. Therefore, we controlled the following social class and background factors in our model: gender, age, education (1 = below secondary; 4 = degree or above), employment status (1 = not working; 2 = working part-time; 3 = working full-time), and residence type (1 = one-room to three-room HDB; 2 = four-room or five-room HDB; 3 = private apartment, condominium or landed house). Recent studies also reported that fathers and mothers may have different perceptions about parenting, and fathers’ involvement in child-rearing is important for children’s health practice [62, 63]. These factors were also controlled for. Father/mother involvement was measured by asking whether mostly father/mother does the five types of household tasks (e.g., planning meals, feeding children, watching over child nutrition, cooking, and ensuring sufficient physical exercise; 1 = Yes and 0 = No).

### Analysis

The primary aim of the present study is to examine the mediating effects of health knowledge on the relationship between social support and health practice. We use structural equation modeling (SEM) via Stata 15.0 to conduct mediation analysis.

*Descriptive analysis*. Before SEM, we conducted descriptive analysis and correlation analysis to preliminarily describe our variables and their relationships. We also used a series of tests to examine the reliability and validity of our measurements. We first examine the reliability of each measurement using ordinal alpha as all the scale items are ordinal and non-normally distributed [64]. An alpha coefficient of 0.70 or higher is usually considered as a cutoff point for good internal consistency but a value between 0.50 and 0.60 is still acceptable for preliminary studies in social sciences, especially for scales with a limited number of items [65–69]. We then exmined composite reliability (CR) and average variance extracted (AVE). CR values ranges from 0.70 to 0.80. and AVE ranges from 0.40 to 0.60. According to Forrell and Lacker [70], AVE values below 0.50 are acceptable if CR is above 0.7. Regarding the diagnosis of convergent and discriminant validity, we followed the methods by Courvoisier et al. and Wingenfeld et al. [71, 72]. It is deemed adequate if within-scale item-to-total correlations are greater than between-scale item-to-total correlations.

*Structural equation modeling (SEM). *To test the mediating effects of BMI and nutrition knowledge in the relationship between parental social support and children’s health practice, structural equation modeling (SEM) via Stata 15.0 was employed. Comparing to traditional mediation analysis through step-by-step regression, SEM has many advantages, especially when models include latent variables and more than one mediator [73]. The SEM package with Stata can directly estimate the indirect effects (mediating effects) of the main predictor which makes the mediation test much easier. SEM also can produce model fit information about the consistency between the data and the hypothesized model.

We used multiple goodness-of-fit indices to assess the model fit [74]: root means squared error of approximation (RMSEA), standardized root means squared residual (SRMR), comparative fit index (CFI), and chi-square to the degree of freedom ratio (χ2/df). Values smaller than 0.1 for RMSEA indicate acceptable fit, and values between 0.05 and 0.08 indicate a good fit [75]. Values less than 0.08 for SRMR show a good fit [76]. Values of CFI greater than 0.90 indicate adequate fit [77]. Scholars also suggested that a value of SBχ2/df lower than 3 indicates a good fit [78].

**Fig. 2:**
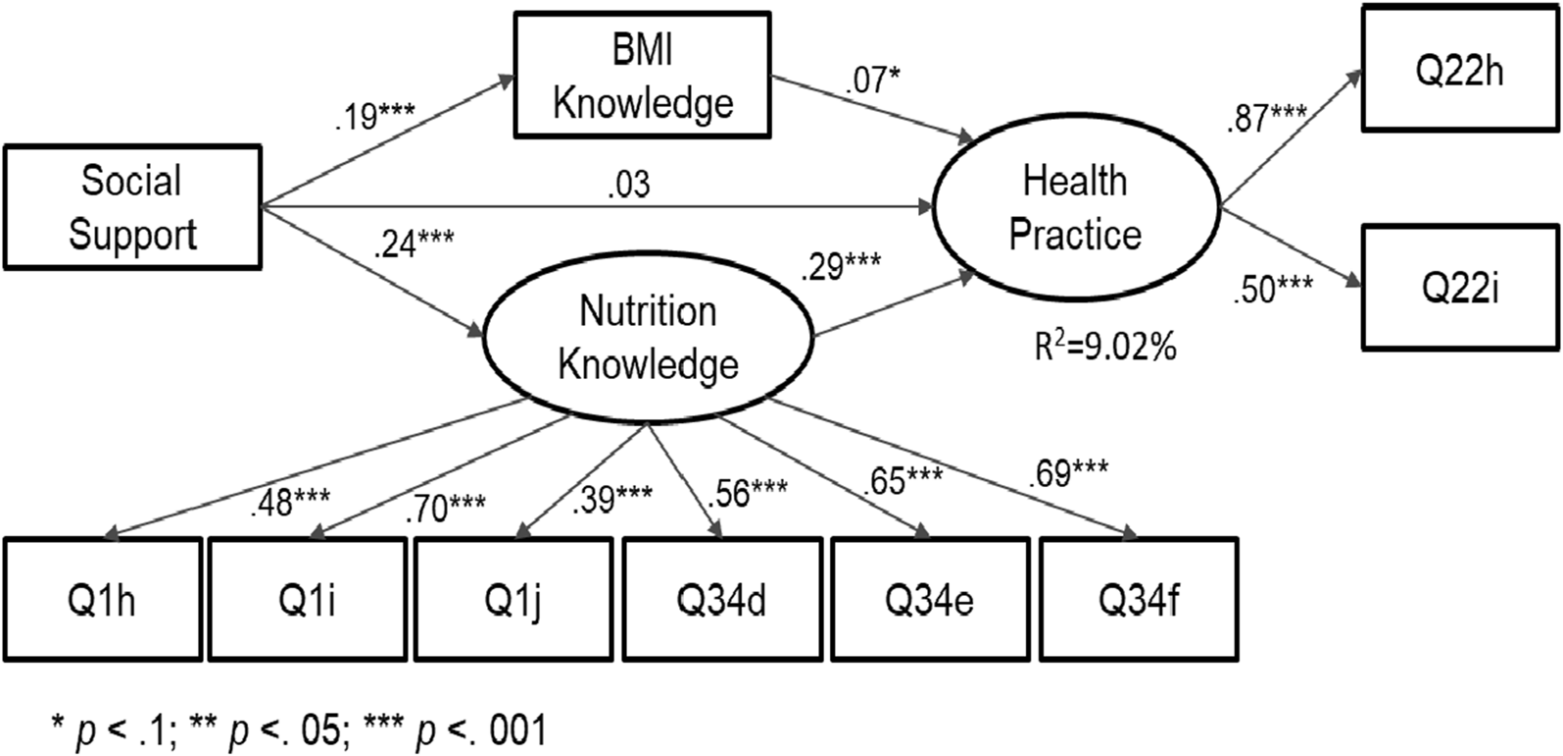
SEM model 1 testing the mediating effects of health knowledge on the relationship between social support and children’s health practice

**Fig. 3:**
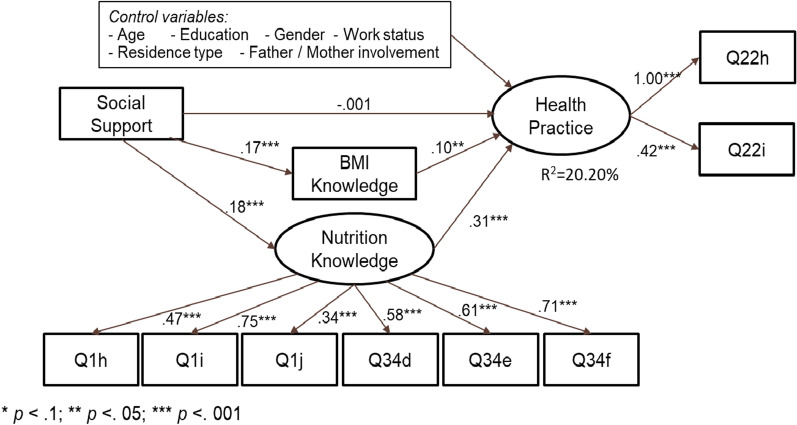
SEM model 1 testing the mediating effects of health knowledge on the relationship between social support and children’s health practice. Parameters of control variables are not presented in this graph due to parsimony consideration

We build two SEM models for comparison purposes. Model 1 (see Fig. 2) contains the predictor (social support), dependent variable (health practice), and two mediators (BMI and Nutrition knowledge). In Model 2 (see Fig. 3), we add additional control variables (e.g., age, gender, education, work status, and mother/father involvement.

## Results

### Descriptive analysis

The results in Table 3 show that respondents have a moderate level of knowledge about BMI and nutrition – out of the 3 questions asked on awareness of what BMI measures, most were able to respond affirmative to 2 out of 3 indicators. On average, children exercise less than once a week, with the mean falling just below the average of once a week (mean = 2.94). Correlations between the dependent variable and key independent variables range from 0.09 to 0.22, thus assuring that there are no issues with collinearity between variables in the model. Nutrition knowledge is positively associated with children’s health practices (*r* = 0.22). Social support is found to have significant positive associations with social support and health knowledge. Gender, age, and education level were included as control variables.

**Table 3:** Descriptive statistics and correlation

Variable	Mean	SD	Min	Max	Ordinal *α*	CR	AVE	1	2	3	4
1. Health practice	2.94	1.13	1	5	0.64	0.71	0.58	–			
2. BMI knowledge	2.22	0.89	0	3	0.72	**0.68**	**0.53**	**0.09**	–		
3. Nutrition knowledge	3.05	0.37	1.67	4	0.70	**0.80**	**0.50**	**0.22**	**0.09**	–	
4. Social Support	4.20	1.21	0	5	0.86	**0.79**	**0.41**	**0.11**	**0.17**	**0.16**	–
5. Gender	–	–	0	1	–			**− **0.04	**− 0.06**	**− 0.11**	**− **0.04
6. Age group	–	–	1	5	–			**0.06**	0.001	− 0.03	**- 0.10**
7. Education	–	–	1	4	–			**0.11**	**0.21**	**0.07**	**0.15**
8. Work status	–	–	1	3	–			**− **0.03	0.05	**− 0.13**	0.001
9. Residence type	–	–	1	3	–			0.01	**0.11**	**− **0.01	0.03
11. Mother’s involvement	2.44	1.81	0	5	–			0.04	0.002	**0.10**	**− **0.06
12. Father’s involvement	0.40	1.01	0	5	–			0.01	**− 0.07**	**− **0.03	**− 0.07**

*N* = 1484. Bold coefficients: *p* < 0.05. *AVE* Average variance extracted. *CR* Composite reliability

Table 3 also shows that ordinal alpha ranges from 0.64 to 0.86 for the four scales, demonstrating acceptable internal reliability of each measurement. CR values ranges from 0.68 to 0.80. and AVE ranges from 0.41 to 0.58. Results of AVE also support our measurement tools’ convergent validity. The convergent and discriminant validity were supported by correlation analysis in Appendix Table 5 showing that within-scale item-to-total correlations are stronger than between-scale item-to-total correlations.

### Structural equation modeling (SEM)

Results of Model 1 and 2 are shown in Figs. 2 and 3 respectively. Results for Model 1 show a good model fit (see Table 4): χ2/*df* = 69.11/30 < 3, RMSEA = 0.039; CFI = 0.958; SRMR = 0.033. All predictors explain 9.02% of the variance in health practice. All the path coefficients through mediators are significant (*p* < 0.1). Results of Model 1 (see Fig. 2) also show that 76.85% of the total effects of social support on health practice are mediated by BMI and nutrition knowledge: Total effect = 0.108 (*p* = 0.008), indirect effect = 0.083 (*p* = 0.000), and direct effect = 0.025 (*p* = 0.575).

**Table 4:** Descriptive measures of the model fit

Model fit indices	Criteria	Obtained value	
		Model 1	Model 2
*χ*^*2*^/*df*	< 3.00	2.30	1.93
Root means squared error of approximation (RMSEA)	< 0.10	0.039	0.038
Comparative fit index (CFI)	> .90	0.958	0.904
Standardized root means squared residual (SRMR)	< 0.08	0.033	0.037

Estimation of Model 2 (Fig. 3) shows a satisfactory model fit (see Table 4): χ2/*df* = 154.08/80 < 3, RMSEA = 0.038, CFI = 0.904, and SRMR = 0.037. All predictors explain 20.20% of the variance in health practice. All the path coefficients through mediators are significant (*p* < 0.1). Most control variables are not significant except age (*β* = – 0.081, *p* = 0.06), father involvement (*β* = 0.088, *p* = 0.07), and residence type (*β* = – 0.074, *p* = 0.09). The total effect of social support is 0.081 (*p* = 0.071), the indirect effect through BMI knowledge and nutrition knowledge is 0.081 (*p* = 0.007), and its direct effect is not significant (*p* > 0.1). This demonstrates that the two types of health knowledge have full mediation effects on the relationship between social support and children’s health practice.

Since Model 2 explains more variance in the dependent variables, we use the results from Model 2 for further reference. According to estimation results from Model 2, all the path coefficients are significant (*p* < 0.001) except the direct path between social support and health practice. Therefore, Hypothesis H2 and H3 are supported. We can also conclude that the mediating effects of BMI and nutrition knowledge on the relationship between parental social support and children’s health practice are supported (Hypothesis H4 is supported). Although the direct path between social support and health practice is not significant due to the full mediation effects (100%), the total effects of social support on health practice in both Model 1 and 2 are significant (*p* = 0.008 and *p* = 0.071). This means that the relationship between social support and health practice is significant and positive when mediators are not included in the model. Thus, Hypothesis H1 is supported (Fig. 3).

## Discussion

### Summary of findings

Using structural equation modeling on a representative sample of Singaporean households with children aged 14 or younger, we found that parents’ social support and health knowledge significantly associate with children’s participation in weight management practices (e.g., physical exercises). More importantly, our results support that parents’ health knowledge serves a mediating role in the relationship between parents’ social support and children’s health practices in weight management. Specifically, there is a significant positive relationship between parents’ social support and health knowledge, thus suggesting that parents draw pro-health information from their social support network. Further, it is noted that the direct effect of social support of parents on children’s health practices is not significant after the mediation effects of health knowledge are considered, which suggests the full mediation effects of health knowledge. These findings contextualize the relationship between social support and health outcomes and advance our theoretical appreciation of the impact of social support as an essential resource. The empirical distillation of the mediation effects advised how pro-health information can be effectively disseminated and will have helpful in framing public health initiatives.

### Theoretical contributions

Three theoretical contributions are noteworthy. First, the present study complements existing knowledge on social determinants (e.g., parents’ social support and health knowledge) of childhood obesity, that there is a direct and positive link between social support and health-related behaviors or outcomes [11, 12]. Our model shows that social support from an individual’s networks does not always necessarily impact pro-health behaviors. As with all peer influence, the normative behaviors of peers vary, as do their credibility as resource persons for health information.

Second, the current study tests a mediation model that bridges social support theories, health knowledge literature, and childhood obesity research. Our findings provide empirical evidence for how children’s health practice is influenced by parents’ social support and health knowledge. The mediating role of health knowledge in the relationship between social support and health practice was supported, which responded to the doubt about why inconsistent findings on the relationship between social support and health practice exist [11–15, 39, 51]. The SEM model demonstrates that while both parents’ BMI and nutrition knowledge fully mediates the relationship between parents’ social support and children’s health practices, compared to BMI knowledge, parents’ nutrition knowledge plays a stronger role.

Finally, this is one of the few studies on the effects of social support on health behavior conducted on an affluent and multi-cultural Southeast Asian population. Although obesity is not traditionally considered a big problem in Asian countries, the growing prevalence of obesity rates attracts increasing attention from researchers and policymakers. Our findings thus contribute to existing knowledge by grounding it within an Asian context.

### Policy values

The findings clarify how pro-health information can be more effectively disseminated to the general public. Health promotion and obesity prevention programs should target participants’ social support networks. Public health messages that are too broad-based and targeted at a general audience dissipate without impacting their target audience. In addition to focusing on parents with young children, our research suggests that another important avenue for disseminating pro-health messages through social support networks, perhaps with simple tag lines like “share this information with a parent of young children”. Against the backdrop of the persistent COVID-19 pandemic, public health educators or governments can be better informed by this study how to guarantee a successful vaccination campaign.

Concurrently, an effort to evolve a network of public health champions in the community may be an effective way of disseminating pro-health information and advisories. These champions can be positioned as support resources to partner parents in their childrearing endeavors. In parenting talks and community education events, invited parents can bring a friend so that information disseminated can reach a larger audience. Such interventions will encourage the provision of social support from sources with higher levels of health knowledge.

One highlight from our study alerted us to the lack of understanding on how the BMI is derived and what it can be used for, and how to make sense of food nutrition labels to support their children’s well-being. This is a reminder that while we have made many advances in pushing out tests and procedures to push out nutrition and health information, for these to impact health practice, we have to invest in educating the lay public on how to render relevance to such information in their everyday practice.

### Limitations and future research directions

Limitations appear in the present study. Our study is based on an analysis of cross-sectional data, which may limit the validity of our results and interpretation. Researchers elsewhere suggest the use of longitudinal rather than cross-sectional data to establish the inference of causality and mediation models [79]. Due to limited resources, the present study was also only able to collect data from parents to test our hypotheses. Information from the child’s position is absent. Future studies may consider a longitudinal research design and collect data based on a parent–child dyad approach. Further, the findings presented in this paper are a small section taken from a more extensive study on sociocultural environmental effects on childhood obesity, and have only limited instruments to measure for health practice and health knowledge. Although we believe that our results based on the current measurement scales are still trustworthy, there is a need to improve the validity and reliability of the scales. Future studies should include more detailed instruments to capture these constructs holistically. For example, the health knowledge should be expanded to include awareness of risk factors of childhood obesity on adult chronic diseases morbidity. Health practice scale should include more information about children’ physical activities and diet management. While this paper demonstrated the effect of access to social support on health knowledge, future research should elaborate on the more complex effects of social support on other aspects of pro-health behaviors.

## Conclusion

The present study aimed to investigate the joint influence of parents’ social support and health knowledge on children’s health practice. Results from our analysis on a nationally representative sample from Singapore support the view that parents’ obesity-related health knowledge has a mediating effect on the relationship between parents’ social support and children’s obesity-related health practice. This indicates that the influence that social support has on health practice is heterogeneous – while parents’ social support has a positive effect on children’s body weight management practices when social support could enhance obesity-related health knowledge, this is not the case when there is a lack of health knowledge embedded in parents’ social support. This study highlighted the family environmental factors of children’s health from the perspective of social support theories, health knowledge literature, and childhood obesity research. Future studies should adopt a longitudinal research design and include more comprehensive instruments to measure the constructs of social support, obesity-related health knowledge, and obesity-related health practices.

## Data Availability

The datasets generated and/or analysed during the current study are not publicly available due to personal privacy of participants, but summary tables may be requested from the corresponding author.

## Appendix

See Table

**Table 5:** Correlations between scale items

		Q32a	Q32b	Q32c	Q32d	Q32e	Q1h	Q1i	Q1j	Q34d	Q34e	Q34f	BMIK1	BMIK2	BMIK3	Q22h	Q22i
Social support	Q32a	1.00															
	Q32b	0.44	1.00														
	Q32c	0.28	0.25	1.00													
	Q32d	0.21	0.21	0.50	1.00												
	Q32e	0.23	0.25	0.30	0.40	1.00											
Nutrition knowledge	Q1h	0.05	0.05	0.04	0.08	0.09	1.00										
	Q1i	0.05	0.04	0.04	0.11	0.10	0.34	1.00									
	Q1j	0.03	0.05	0.08	0.09	0.14	0.23	0.25	1.00								
	Q34d	0.01	0.03	– 0.04	0.00	0.03	0.16	0.14	0.14	1.00							
	Q34e	0.04	0.03	0.02	0.04	0.06	0.16	0.19	0.17	0.40	1.00						
	Q34f	0.10	0.09	0.09	0.13	0.18	0.16	0.25	0.19	0.33	0.45	1.00					
BMI knowlede	BMIK1	0.11	0.10	0.18	0.12	0.21	0.07	0.08	0.14	0.05	0.00	0.12	1.00				
	BMIK2	0.14	0.14	0.20	0.14	0.23	0.07	0.05	0.12	0.06	0.01	0.06	0.47	1.00			
	BMIK3	0.02	0.05	– 0.01	0.03	0.03	0.02	0.05	0.05	0.02	– 0.03	– 0.03	0.22	0.12	1.00		
Health practice	Q22h	0.11	0.09	0.05	0.05	0.12	0.15	0.16	0.10	0.10	0.09	0.14	0.11	0.12	0.05	1.00	
	Q22i	0.06	0.04	0.02	0.05	0.11	0.09	0.08	0.07	0.13	0.07	0.15	0.08	0.09	– 0.01	0.47	1.00

^5^
